# Mouse *Sox17* haploinsufficiency leads to female subfertility due to impaired implantation

**DOI:** 10.1038/srep24171

**Published:** 2016-04-07

**Authors:** Yoshikazu Hirate, Hitomi Suzuki, Miyuri Kawasumi, Hinako M. Takase, Hitomi Igarashi, Philippe Naquet, Yoshiakira Kanai, Masami Kanai-Azuma

**Affiliations:** 1 Department of Experimental Animal Model for Human Disease, Graduate School of Medical and Dental Sciences, Tokyo Medical and Dental University (TMDU), Bunkyo-ku, Tokyo, 113-8510, Japan; 2 Center for Experimental Animals, TMDU, Bunkyo-ku, Tokyo, 113-8510, Japan; 3 Department of Veterinary Anatomy, The University of Tokyo, Bunkyo-ku, Tokyo, 113-8657, Japan; 4 Centre d’Immunologie de Marseille-Luminy, Aix Marseille Université UM2, Inserm, U1104, CNRS UMR7280, 13288 Marseille, France

## Abstract

Embryonic implantation comprises a dynamic and complicated series of events, which takes place only when the maternal uterine endometrium is in a receptive state. Blastocysts reaching the uterus communicate with the uterine endometrium to implant within a narrow time window. Interplay among various signalling molecules and transcription factors under the control of ovarian hormones is necessary for successful establishment of pregnancy. However, the molecular mechanisms that allow embryonic implantation in the receptive endometrium are still largely unknown. Here, we show that Sry-related HMG box gene-17 (*Sox17*) heterozygous mutant female mice exhibit subfertility due to implantation failure. Sox17 was expressed in the oviduct, uterine luminal epithelium, and blood vessels. *Sox17* heterozygosity caused no appreciable defects in ovulation, fertilisation, blastocyst formation, and gross morphology of the oviduct and uterus. Another group F Sox transcription factor, Sox7, was also expressed in the uterine luminal and glandular epithelium relatively weakly. Despite uterine Sox7 expression, a significant reduction in the number of implantation sites was observed in *Sox17* heterozygous mutant females due to haploinsufficiency. Our findings revealed a novel role of Sox17 in uterine receptivity to embryo implantation.

Embryonic implantation in the uterus is an essential event that allows progression of embryogenesis beyond the blastocyst stage and takes place during the fifth day of development in mice^1^. Blastocysts reach the uterus, hatch from the zona pellucida, and thereafter implant into the uterus, only when the uterus is in a receptive state. The uterus is composed of endometrium and myometrium. Of these, the endometrium dynamically changes during pregnancy. Invagination of the luminal epithelium occurs at the antimesometrial side to form a crypt structure, wherein the blastocyst settles. Following blastocyst attachment, decidualisation of the surrounding stromal cells takes place, allowing the embryos to develop further. In humans and rodents, uterine receptivity is primarily coordinated by the ovarian hormones progesterone and estrogen^1^,^2^. Under the influence of these hormones, interplay between signalling molecules and transcription factors orchestrates these dynamic events, leading to successful embryonic implantation^1^.

Sry-related HMG box gene 17 (Sox17), together with Sox7 and Sox18 comprises the group F Sox proteins^3^. These proteins share conserved amino acid sequences in the DNA-binding high mobility group (HMG) box domain and play similar roles in several developmental events, such as cardiovascular development^4^,^5^,^6^,^7^,^8^, haematopoiesis^9^,^10^, endoderm formation^11^, and human primordial germ cell specification^12^. Sox17 is expressed in the uterine luminal epithelium during the implantation period with an elevated level at the embryo attachment site^13^. However, the role of Sox17 in implantation has not been addressed in previous studies. In this study, we showed that Sox17 was expressed in the oviduct and uterine luminal and glandular epithelium and that *Sox17* heterozygous mutant females exhibited subfertility due to implantation failure. The results from this study suggested a novel role of Sox17 in uterine receptivity and embryonic implantation.

## Results

### Sox17 was expressed in female reproductive organs

**Figure 1:**
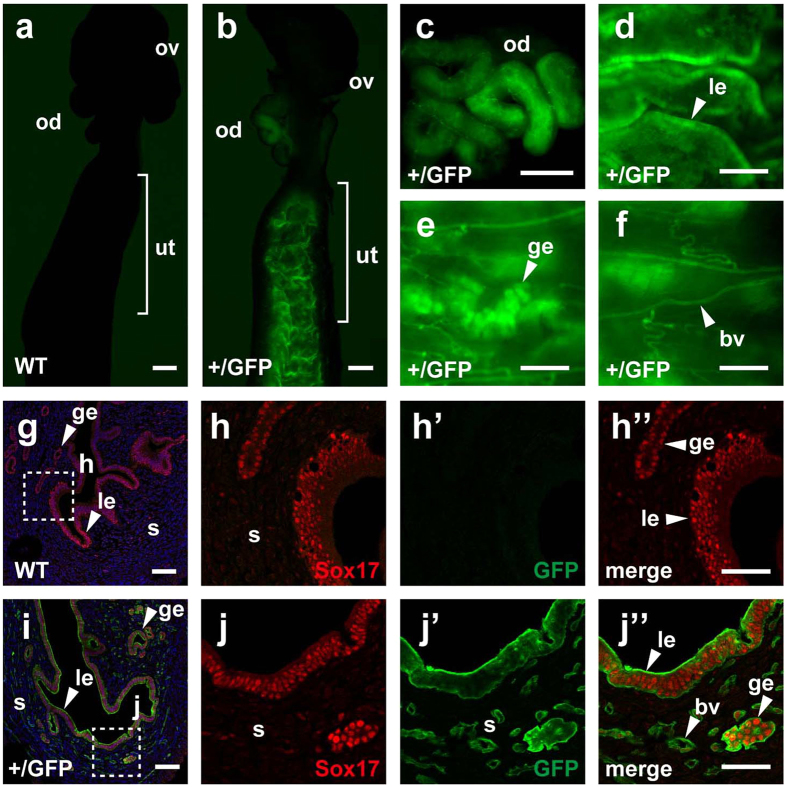
Expression of Sox17 in the oviduct and uterus. (**a–f**) Fluorescence emitted from GFP without staining in nonpregnant, WT and *Sox17*^+/GFP^ female reproductive organs. (**g–j”**) Immunofluorescent staining of GFP (green) and Sox17 (red) in the WT (**g–h”**) and *Sox17*^+/GFP^ (**i–j”**) uterus. Nuclei are stained with Hoechst (blue). Enlarged images corresponding to dotted boxes in (**g**) and (**i**) are shown in (**h–h”**) and (**j–j”**), respectively. Abbreviations: ov, ovary; od, oviduct; ut, uterus; le, luminal epithelium; ge, glandular epithelium; s, stroma; and bv, blood vessels. Scale bars, 500 μm for (**a–c**); 200 μm for (**d–f**); 100 μm for (**g**,**i**); and 50 μm for (**h”**,**j”**).

To examine *Sox17* expression in female reproductive organs, we first used *Sox17*-green fluorescent protein (GFP) knock-in mice, which express GFP under the control of the *Sox17* promoter^9^. In nonpregnant, *Sox17*-GFP heterozygous mutant females (*Sox17*^+/GFP^), GFP fluorescence was observed in the oviduct and uterine endometrium (Fig. 1b–d), whereas wild-type (WT) female mice showed no discernible fluorescence in these organs under the same conditions of image acquisition (Fig. 1a). In the uterine endometrium, both the luminal and glandular epithelium expressed GFP (Fig. 1d,e). Blood vessels also expressed GFP (Fig. 1f). Uterine expression of GFP was stronger than that observed in the blood vessels.

To examine whether uterine expression of GFP in *Sox17*^+/GFP^ mice coincided with that of endogenous Sox17, we performed co-immunofluorescent staining using anti-Sox17 and anti-GFP antibodies. In the WT uterus, Sox17 staining was detected in the luminal and glandular epithelium (Fig. 1g–h”). In the *Sox17*^+/GFP^ uterus, GFP staining coincided with Sox17 staining (Fig. 1i–j”), confirming the reliability of Sox17-GFP for monitoring Sox17 expression. Fluorescence emitted from GFP without staining was also detectable (Supplementary Fig. S1). However, GFP staining was more distinct and clearly showed GFP-positive tubular structures in the stroma (Fig. 1j–j”). These Sox17-GFP-positive cells were likely to be capillary blood vessels. These microscopic analyses suggest that Sox17 was expressed in the oviduct and uterine luminal epithelium, with weak expression in the blood vessels of nonpregnant, female mice. Importantly, *Sox17*^+/GFP^ mice had no appreciable defects in uterine morphology, including the blood vessels.

**Figure 2:**
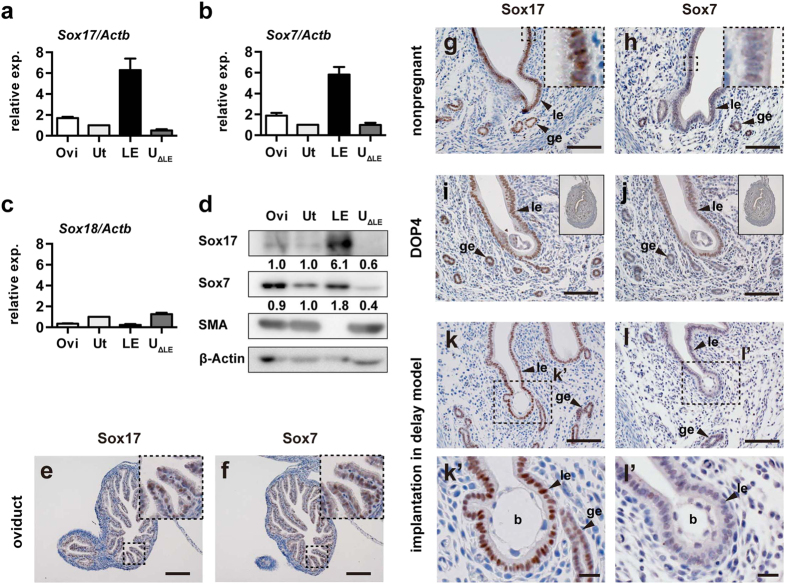
Expression of Sox17, Sox7, and Sox18 in the oviduct and uterus receptive to implantation. (**a–c**) Relative expression levels of *Sox17* (**a**), *Sox7* (**b**), and *Sox18* (**c**) transcripts in WT oviduct and uterine tissues at 18:00–20:00 on DOP 4 were determined by real-time RT-PCR. Relative expression levels (2^−ΔΔCT^) were analysed by the comparative C_T_ method (mean ± standard error of mean [SEM]). *Actb* was used as an internal control. The uterus was used as a calibrator. (**d**) Western blot analysis to show Sox17 and Sox7 protein levels in the WT oviduct and uterine tissues prepared at DOP 4 simultaneously with RNA samples. Lack of smooth muscle actin (SMA) expression indicates proper preparation of the luminal epithelium. β-Actin was used as a loading control. The numbers beneath the Sox17 and Sox7 bands represent relative signal levels (Ut = 1.0), in which β-Actin was used for normalisation. (**e,f**) Immunohistochemistry of Sox17 and Sox7 in the WT oviduct at DOP 4. Enlarged images corresponding to the dotted boxes are shown in insets. (**g**,**h**) Immunohistochemistry of Sox17 and Sox7 in nonpregnant, WT uteri. Enlarged images corresponding to the small dotted boxes are shown in insets. (**i,j**) Immunohistochemistry of Sox17 and Sox7 in the WT uteri at DOP 4. Low magnification images of the cross-sectioned uteri are shown in insets. (**k–l’**) Immunohistochemistry of Sox17 and Sox7 in the WT uteri at 6 h after administration of P4 + E2 in the delayed implantation model. Enlarged images corresponding to dotted boxes in (**k**,**l**) are shown in (**k’**,**l’**), respectively. Abbreviations: Ovi, oviduct; Ut, uterus; LE(le), luminal epithelium; U_ΔLE_, uterine tissues after removal of LE; ge, glandular epithelium; and b, blastocyst. Scale bars, 100 μm for (**e–l**); and 20 μm for (**k’**,**l’**).

Next, we examined the expression of Sox17 together with other Sox-F group transcription factors, Sox7 and Sox18, in the oviduct, uterus, and uterine tissue fractions at day-of-pregnancy (DOP) 4 in natural pregnancy. Here, DOP 1 was the day at which the vaginal plug was formed, and DOP 4 was the day at which the uterus became receptive to implantation. First, we performed quantitative reverse transcription polymerase chain reaction (RT-PCR), in which *Actb* was used as an internal control, and the uterus was used as a calibrator (Fig. 2a–c). Similar results were also obtained using *Gapdh* as an internal control (Supplementary Fig. S2). In the oviduct, *Sox17, Sox7*, and a lesser amount of *Sox18* transcripts were detected; the expression levels were 1.7-, 1.9-, and 0.3-fold higher, respectively, than those detected in the uterus (n = 5; Fig. 2a,b). In the uterine luminal epithelium, *Sox17* and *Sox7* transcripts were abundant, exhibiting 6.3- and 5.8-fold higher expression, respectively, than that observed in the uterus (n = 5; Fig. 2a,b). In contrast, *Sox18* transcript levels in the luminal epithelium were negligible (Fig. 2c). Moreover, *Sox18* transcripts were undetectable in one of the five samples from the luminal epithelium.

Next, we performed western blotting to examine Sox17 and Sox7 protein levels. Proper preparation of the luminal epithelium was ensured by absence of smooth muscle actin (Fig. 2d). Similar to the PCR analysis, the blot was quantified by normalisation with β-actin and divided by the value from the uterus (i.e., uterine expression = 1.0). In the quantification, the amounts of Sox17 and Sox7 proteins were 6.1- and 1.8-fold higher than those observed in the uterus. These data implied that, among the Sox-F proteins, Sox17 was mainly expressed in the luminal epithelium.

To examine the tissues expressing Sox17 and Sox7 in the oviduct and uterus, we conducted immunohistochemistry (Fig. 2e–l’). In the oviduct, staining for both Sox17 and Sox7 was clearly detected at a comparable level (Fig. 2e,f). In the nonpregnant WT uterus, staining for Sox17 and Sox7 was found in the luminal and glandular epithelium (Fig. 2g,h). Consistent with their function as transcription factors, the staining for Sox17 and Sox7 was localised exclusively in the nuclei (insets in Fig. 2g,h). Similar patterns of Sox17 and Sox7 staining were also observed at the implantation site in the DOP 4 uteri after natural mating (Fig. 2i,j, and Supplementary Fig. S1) and in a delayed implantation model (Fig. 2k,l). The latter was used to ensure the timing of implantation. Consistent with a previous report^13^, Sox17 showed moderately elevated expression at the embryo attachment site (Fig. 2k,k’). These data suggested that Sox17 and Sox7 were expressed in the oviduct and uterine luminal epithelium receptive to implantation.

### *Sox17* heterozygous mutant females exhibit subfertility

**Figure 3:**
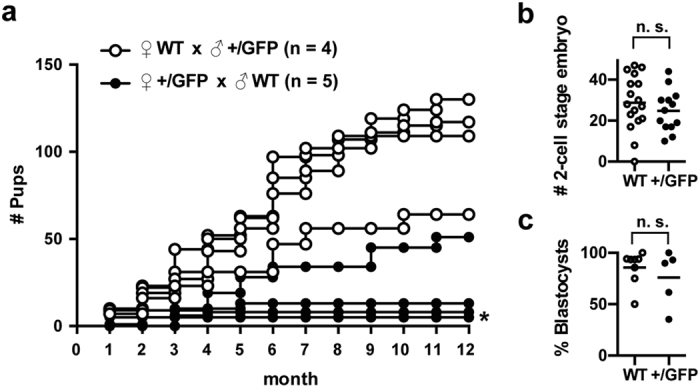
Subfertility of *Sox17*^+/GFP^ female mice. (**a**) Cumulative number of pups from WT females mated with *Sox17*^+/GFP^ males (○) (n = 4) and from *Sox17*^+/GFP^ females mated with WT males (●) (n = 5). (**b**) Number of 2-cell stage embryos collected from WT and *Sox17*^+/GFP^ females after superovulation treatment. (**c**) Percent of blastocysts that were developed normally from 2-cell stage embryos collected from WT and *Sox17*^+/GFP^ females after mating with WT males. Abbreviation: n.s., not significant. An asterisk (*) shows two lines that are completely overlapped.

While breeding Sox17-GFP mice, we noticed that the number of pups from *Sox17*^+/GFP^ female and WT male pairs was fewer than that from WT female and *Sox17*^+/GFP^ male pairs. For quantitative analysis, we counted the cumulative number of pups while breeding Sox17-GFP mice for 12 months (Fig. 3a). WT female and *Sox17*^+/GFP^ male pairs produced an average of 105.0 ± 14.3 pups (n = 4), whereas *Sox17*^+/GFP^ female and WT male pairs produced an average of 16.4 ± 8.8 pups (n = 5). These numbers include the pups that died soon after birth. The difference was statistically significant as determined by *t*-tests (*p* = 0.0141). Notably, *Sox17*^+/GFP^ females had a smaller litter size and tended to become infertile early in adulthood.

Since *Sox17* haploinsufficiency results in perinatal lethality in C57BL/6 background^14^, we also examined the genotype of the pups after weaning. WT to +/GFP ratio was 161:139 for the pups produced by WT females and 39:37 for the pups produced by *Sox17*^+/GFP^ females. Chi-square test revealed that WT and +/GFP pups were born approximately at the expected Mendelian ratio of 1:1 for both the mating groups (*p* = 0.204 and 0. 815 from WT and *Sox17*^+/GFP^ females, respectively), indicating that perinatal lethality of *Sox17*^+/GFP^ pups was not a major reason for the subfertility observed in *Sox17*^+/GFP^ females.

Subsequently, to examine the influence of *Sox17* heterozygosity on the ovulation, fertilisation, and early development, we collected 2-cell stage embryos from superovulated, WT and *Sox17*^+/GFP^ females and found that the number of the embryos was comparable (Fig. 3b). These 2-cell stage embryos were further cultured *in vitro* and formed blastocysts at a similar rate (Fig. 3c). These data suggested that ovulation, fertilisation and embryonic development to the blastocyst stage were phenotypically normal and were not affected by *Sox17* heterozygosity.

### *Sox17* heterozygous mutant females were defective in implantation

**Figure 4:**
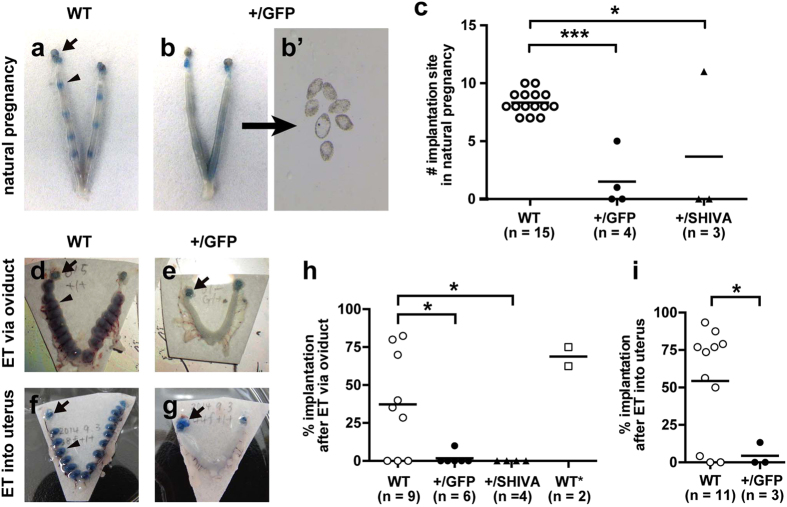
Defective implantation in *Sox17* heterozygous mutant females. (**a,b**) Blue dye staining showing implantation sites in WT (**a**) and *Sox17*^+/GFP^ (**b**) uteri at DOP 5 after natural mating. Arrows and arrowheads show the ovaries and implantation sites, respectively. (**b’**) Unimplanted blastocysts recovered from *Sox17*^+/GFP^ uterus by flushing. (**c**) Number of implantation site in females of the indicated genotype at DOP 5 after natural mating. Dots and bars show sample and mean values, respectively. (**d–g**) Blue dye staining showing implantation sites in WT (**d,f**) and *Sox17*^+/GFP^ (**e,g**) uteri at DOP 7 after embryo transfer (ET). WT embryos were transferred either to the oviduct (**d**,**e**) or the uterus (**f**,**g**). (**h**,**i**) Implantation rates at DOP 6 or 7 in embryo-transferred females of the indicated genotype. WT embryos were transferred via the oviduct (**h**) or in the uterus (**i**), except for WT*, to which approximately a 1:1 mixture of WT and *Sox17*^+/GFP^ embryos were transferred. *p < 0.05; **p < 0.01; ***p < 0.001 as per one-way analysis of variance Dunnett’s multiple comparisons test (**c**,**h**) and *t*-tests (**i**).

To investigate when the embryos were lost in *Sox17*^+/GFP^ females, we counted the number of implantation sites at DOP 5, which was the day blastocysts were implanted into the uterus. WT females showed an average of 8.3 ± 0.3 (n = 15) implantation sites (Fig. 4a,c). In contrast, *Sox17*^+/GFP^ females showed an average of 1.5 ± 1.2 (n = 4) implantation sites (Fig. 4b,c). The difference was statistically significant (*p* < 0.0001 by *t*-tests). Notably, unimplanted blastocysts were recovered from the *Sox17*^+/GFP^ uteri with no implantation sites (Fig. 4b’), clearly indicating the normal function of the oviduct and normal development of the blastocyst. Since GFP expressed in *Sox17*^+/GFP^ female mice may exert adverse effects on the number of implantation sites, we also counted the number of implantation sites using Sox17 point-mutant mice (*SHIVA*)^15^, with a point mutation that alters the 72^nd^ Met to Ala in the DNA-binding region of the HMG domain. *Sox17*^+/SHIVA^ female mice showed an average of 3.7 ± 3.7 implantation sites (n = 3; *p* = 0.0077 by *t*-tests; Fig. 4c), confirming that GFP expression was not responsible for the implantation failure observed in *Sox17*^+/GFP^. These results indicated that implantation was defective in *Sox17* heterozygous mutant females.

To directly demonstrate the failure in implantation, we performed embryo transfer and examined the implantation rate. In embryo transfer, we used WT embryos to exclude the possibility that the implantation failure was caused by problems with the embryo. In addition, the implantation site was observed at DOP 6 or 7 to examine whether the smaller number of implantation sites observed at DOP 5 in *Sox17* heterozygous mutant females could be explained by factors other than a delay in implantation.

First, we transferred WT embryos into the oviduct. WT female mice showed an average implantation rate of 37.4% ± 11.3% (n = 9; Fig. 4d,h). In contrast, *Sox17*^+/GFP^ female mice showed a much lower average implantation rate (1.7% ± 1.7%, n = 6; Fig. 4e,h). The difference was statistically significant (*p* = 0.0273 by *t*-tests). *Sox17*^+/SHIVA^ female mice showed no implantation sites (n = 4; Fig. 4h). We also transferred approximately a 1:1 mixture of WT and *Sox17*^+/GFP^ embryos to WT female mice to examine whether *Sox17*^+/GFP^ embryos could be implanted successfully. Female mice with transferred embryos showed an average implantation rate of 68.8% ± 6.3% (n = 2). This number was comparable to the results of WT embryo transfer, suggesting that *Sox17* heterozygosity in embryos was not responsible for implantation failure. This was also supported by the normal number of pups from the mating of WT female and *Sox17*^+/GFP^ male mice (Fig. 3a).

Secondly, we transferred WT embryos directly to the uterus. WT females showed an average implantation rate of 54.3% ± 10.9% (n = 11; Fig. 4f,i), whereas *Sox17*^+/GFP^ females showed a much lower average implantation rate (4.4% ± 4.4%, n = 3; Fig. 4g,i). This difference was statistically significant (*p* = 0.0394 by *t*-tests). These results suggested that the smaller number of implantation sites was not due to delays in implantation and further suggested that embryonic implantation into the uterus was defective in *Sox17* heterozygous mutant females.

## Discussion

In this study, we showed expression of Sox17 in the oviduct and uterine luminal and glandular epithelium, female subfertility, and decreased numbers of implantation sites in *Sox17* heterozygous mutant females. In addition to Sox17, we also found expression of Sox7 in the uterine endometrium. However, the fact that most of the *Sox17* heterozygous mutant females showed subfertility and implantation failure and that Sox17 protein was predominant in the luminal epithelium led to the conclusion that Sox17 was a major player in embryonic implantation among Sox-F proteins.

The observed implantation failure may have been caused by haploinsufficiency of the *Sox17* gene. *Sox17* is known to exhibit haploinsufficiency in bile duct formation, which results in biliary atresia and hepatitis in C57BL/6 background mice^14^. This *Sox17* haploinsufficiency may result from lack of redundancy by other Sox-F proteins in the gallbladder and bile duct epithelia. It seems that *Sox17* exhibits haploinsufficiency in implantation in a similar manner. However, we also observed that some *Sox17* heterozygous mutant females had relatively normal litter sizes in mating and numbers of implantation sites. Functional redundancy among the Sox-F transcription factors has been reported in various events, namely, early cardiovascular development^6^,^7^, postnatal angiogenesis^5^, and haematopoiesis^9^,^10^ in mice. In addition, Sox7 and Sox17 modify the *Sox18*-null phenotype in the lymphatic vasculature in a strain-specific manner^16^. In a similar way, it is possible that compensatory increase in Sox7 and/or Sox18 expression may occur in *Sox17* heterozygous mutant females depending on the genetic background. Because we used a hybrid of C57BL/6 and ICR mice in this study, there may be individual differences in genetic background; this may be one reason for the presence of some outliers among the mutants.

Role of Sox17 in implantation remained elusive. However, there are some clues to how Sox17 may function during implantation. ChIP-Seq analysis revealed Sox17 as a direct transcriptional target of the progesterone receptor in the mouse uterus^17^, suggesting the role of Sox17 as a mediator of progesterone functions in early pregnancy. In addition to the progesterone, Leukaemia inhibitory factor (LIF) is an essential factor for implantation^18^. LIF regulates expression of a number of genes critical for implantation, such as the muscle segment homeobox (Msh) gene family member *Msx1* and *Indian hedgehog*^19^,^20^,^21^,^22^. *Sox17* is also reported as one of the genes that are upregulated after LIF treatment^21^. Therefore, progesterone together with LIF is likely to induce *Sox17* expression at the timing of implantation. Intriguingly, Sox17 showed moderately enhanced expression at the blastocyst attachment site (Fig. 2i,k’ and Wallingford *et al*.^13^), implicating the direct role of Sox17 in blastocyst attachment. Of interest, Wnt/β-catenin signalling is also active at the implantation site^23^. Previous studies have reported that Wnt/β-catenin signalling induces Sox17^24^,^25^; moreover, interaction of β-catenin with Sox17 may enhance the activity of Sox17 for induction of its target genes^26^. Therefore, expression of Sox17 together with Wnt/β-catenin signalling may be critical for embryonic implantation.

The mechanism of embryonic implantation is diverse across mammalian species, including humans and mice^27^,^28^,^29^. For example, mice produce many offspring in a single birth, whereas humans produce a single offspring in a single birth; additionally mouse implantation is eccentric (i.e., the blastocyst lies in a uterine crypt), whereas human implantation is interstitial (i.e., the blastocyst is completely embedded within the endometrium)^27^. However, availability of sophisticated genetic engineering has made the mouse an attractive model for human implantation and contributed to reveal the molecular basis of implantation, including elucidation of LIF as a critical factor for implantation^18^,^30^. Evidence from human studies suggests that Sox17 may also play a role in implantation. In a public database “The Human Protein Atlas”^31^,^32^, RNA expression of *SOX17* in the endometrium is reported at a medium level (19 Fragments Per Kilobase gene model and Million reads [FPKM] in RNA-Seq), whereas RNA expressions of *SOX7* and *SOX18* in the endometrium are at low levels (4 and 2 FPKM, respectively). Therefore, it is suggested that SOX17 is the principal Sox-F protein expressed in the human uterus.

Another line of evidence implicates the involvement of *SOX17* in successful pregnancy in humans. In exogenously administrated hormone-stimulated cycles aimed to conduct *in vitro* fertilisation and embryo transfer in humans, advanced endometrial maturation in histological dating compared to the expected chronological date is usually observed, in which, advanced maturation exceeding 3 days never results in successful pregnancy^33^,^34^. Microarray analysis revealed that *SOX17* is one of the genes upregulated in the endometrium with 2–3 days advanced maturation (i.e., possible to achieve pregnancy) compared to those with 4 days advanced maturation (i.e., unable to achieve pregnancy)^35^, suggesting the relevance of SOX17 to pregnancy.

In this study, we found a novel role of uterine Sox17 in embryonic implantation by investigating both bulk knockout (*Sox17*^+/GFP^) and a point mutant (*Sox17*^+/SHIVA^). Further studies of changes in the localisation, expression, and molecular interactions of uterine Sox17 during the estrus cycle and pregnancy and the use of conditional knockout in the luminal epithelium will lead to clarification of the role of uterine Sox17 in implantation and ultimately contribute to improve infertility treatment in humans.

## Methods

### Animals and ethical statement

*Sox17 GFP* knock-in mice (*Sox17*^tm1Sjm^)^9^, and mutant mice carrying a point mutation in the *Sox17* locus (*SHIVA*)^15^ were used in this study. *Sox17*^+/GFP^ mice in the C57BL/6 background are also known to exhibit haploinsufficiency in bile duct formation, which leads to perinatal death due to biliary atresia and hepatitis^14^. For this reason, we used *Sox17* *GFP* knock-in mice in a mixed background of C57BL/6 and ICR. Mice were housed in environmentally controlled, specific pathogen-free rooms in the Center for Experimental Animals of Tokyo Medical and Dental University (TMDU). All experiments were carried out in accordance with the approved guidelines by the institutional committees for animal and recombinant DNA experiments at TMDU. All experimental protocols were approved by the Institutional Animal Care and Use Committee of TMDU (Nos 0130082C, 0140007A, 0150259C2, and 0160024C2).

### Superovulation and *in vitro* culture of embryos

Female mice of 8- to 12-week old were superovulated according to the standard procedure^36^. Pregnant mare serum gonadotropin (PMSG) and human chorionic gonadotropin (hCG) were obtained from ASKA pharmaceutical (Tokyo, Japan) and used at a concentration of 7.5 IU, each. Two-cell stage embryos were collected according to the standard procedure^36^, and cultured at 37 °C, 5% CO_2_ in a drop of KSOM medium (ARK Resource, Kumamoto, Japan) covered with a layer of liquid paraffin (Nacalai Tesque, Kyoto, Japan).

### Implantation site counting

Female mice were naturally mated with male mice. For females with vaginal plug, implantation sites were visualised by intravenous injection of 1% solution of Chicago sky blue dye (Sigma-Aldrich, MO, USA) at 13:00–17:00 on DOP 5^36^.

### Embryo transfer and implantation rate

Female mice were mated with vasectomised males to induce pseudopregnancy. At DOP 1, cryopreserved embryos at the pronuclear or 2-cell stage were thawed and transferred to the oviduct^36^. Alternatively, embryos at the morula or blastocyst stage were transferred to the uterus at DOP 3^36^. The implantation rate was calculated as the number of implantation sites divided by the number of transferred embryos × 100 (%).

### Delayed implantation

Delayed implantation was performed as previously described by Ma *et al*.^37^. Embryo transfer and ovariectomy were conducted at DOP 2. Progesterone (P4) (Sigma-Aldrich) was administrated at DOP 5 and 6. At DOP 7, both P4 and estrogen (E2) (Sigma-Aldrich) was administrated to induce implantation. The uteri were fixed at 6 h after P4 + E2 administration.

### Preparation of the luminal epithelium

The luminal epithelium at DOP 4 was prepared by incubating the uteri in Hank’s balanced salt solution with pancreatin (Sigma-Aldrich) and dispase (Thermo Fisher, MA, USA) at 4 °C for 1 h and successively at room temperature for 1 h.

### Quantitative RT-PCR

Total RNA was prepared from the naturally mated C57BL/6 female mice at 18:00–20:00 on DOP 4 using TRIzol^®^ in combination with a PureLink^®^ RNA mini kit and on-column DNase digestion (Thermo Fisher). Reverse transcription was performed using a SuperScript^®^ III first-strand synthesis system for RT-PCR (Thermo Fisher) with random hexamers. Real-time PCR was performed by StepOne™ with TaqMan^®^ Fast Advanced Master Mix (Thermo Fisher). The TaqMan^®^ gene expression assays used in this study included Sox17 (Mm00488363_m1), Sox7 (Mm00776876_m1), Sox18 (Mm00656049_gH), Actb (Mm02619580_g1), and Gapdh (Mm99999915_g1). Data were analysed by the comparative C_T_ (ΔΔC_T_) method, in which *Actb* or *Gapdh* served as an internal control, and uterus data were used as a calibrator^38^. We confirmed that the target genes and the internal control genes had similar PCR efficiencies, which were close to 100% (Supplementary Fig. S2).

### Western blotting

Protein samples were prepared from the naturally mated C57BL/6 female mice at 18:00–20:00 on DOP 4, separated by sodium dodecyl sulphate polyacrylamide gel electrophoresis (SDS-PAGE) on 10% gels, and transferred to polyvinylidene fluoride (PVDF) membranes. The primary antibodies used in this study included goat anti-Sox17 (R&D Systems, AF1924; 1:500), goat anti-Sox7 (R&D Systems, AF2766; 1:500), mouse anti-α-smooth muscle actin (Sigma-Aldrich, A2547; 1:20,000), and mouse anti-β-actin (Sigma-Aldrich, A1978, 1:2,000). The secondary antibodies used in this study included horseradish peroxidase (HRP)-conjugated donkey anti-goat IgG (Jackson ImmunoResearch, PA, USA) and HRP-conjugated goat anti-mouse IgG (Jackson ImmunoResearch). Amersham™ ECL Select™ (GE Healthcare, IL, USA) was used for detection.

### Tissue processing

Animals were fixed by perfusion of 4% paraformaldehyde (PFA)/phosphate-buffered saline (PBS) after blood removal and female reproductive organs were excised. For observation of whole-mount fluorescence, organs were placed in PBS and photographed by Axio Zoom V16 microscope (Carl Zeiss). For immunostaining, the uteri were further fixed in 4% PFA at 4 °C overnight and embedded in paraffin or optimal cutting temperature (OCT) compound (Sakura Finetek Japan, Tokyo, Japan). After blocking with Tris-NaCl-blocking (TNB) buffer (Perkin Elmer, MA, USA), sections were incubated with primary antibodies diluted with PBS containing 0.1% Triton X-100 or Can Get Signal^®^ solution A (Toyobo, Osaka, Japan). The primary antibodies and dilutions used in this study included chicken anti-GFP (Abcam, ab13970; 1:1,000), goat anti-Sox17 (R&D Systems, AF1924; 1:500), and goat anti-Sox7 (R&D Systems, AF2766; 1:500)^7^,^39^. Secondary antibodies included Alexa 488-conjugated donkey anti-chicken IgG, Cy3-conjugated bovine anti-goat IgG, and Biotin-SP donkey anti-goat IgG (Jackson ImmunoResearch). Heat-induced antigen retrieval in sodium citrate was conducted for immunohistochemistry of paraffin sections. For counterstaining, Meyer’s Haematoxylin (Wako Pure Chemical Industries, Osaka, Japan) or Hoechst 33258 were used. Specimens were mounted with Permount^®^ (FALMA, Tokyo, Japan) or ProLong^®^ Gold antifade reagent (Thermo Fisher) and photographed by BX53 microscope equipped with DP80 camera (Olympus) or by SP8 confocal microscope (Leica microsystems).

### Statistical analysis

All statistical analyses were conducted using Prism 6 software (GraphPad Software, CA, USA). For all analyses, *p* < 0.05 was considered statistically significant. In implantation site counting, females with no implantation site due to failure in ovulation, fertilisation, and embryonic development were excluded from the statistical analysis.

## Additional Information

**How to cite this article**: Hirate, Y. *et al*. Mouse *Sox17* haploinsufficiency leads to female subfertility due to impaired implantation. *Sci. Rep*. **6**, 24171; doi: 10.1038/srep24171 (2016).

## Supplementary Material

Supplementary Information
