# Supplementary material for: Mouse Sox17 haploinsufficiency leads to female subfertility due to impaired implantation
Source: Sci Rep. 2016 Apr 7;6:24171. doi: 10.1038/srep24171 (PMC4823729; doi:10.1038/srep24171)
Supplement: Supplementary Information [file srep24171-s1.pdf]

Supplementary Information for:

Mouse *Sox17* haploinsufficiency leads to female subfertility due to impaired implantation

Yoshikazu Hirate[1,2,a], Hitomi Suzuki[1,a] Miyuri Kawasumi[1,2,a], Hinako M. Takase[1], Hitomi Igarashi[3], Philippe Naquet[4], Yoshiakira Kanai[3], and Masami Kanai-Azuma[1,2,*]

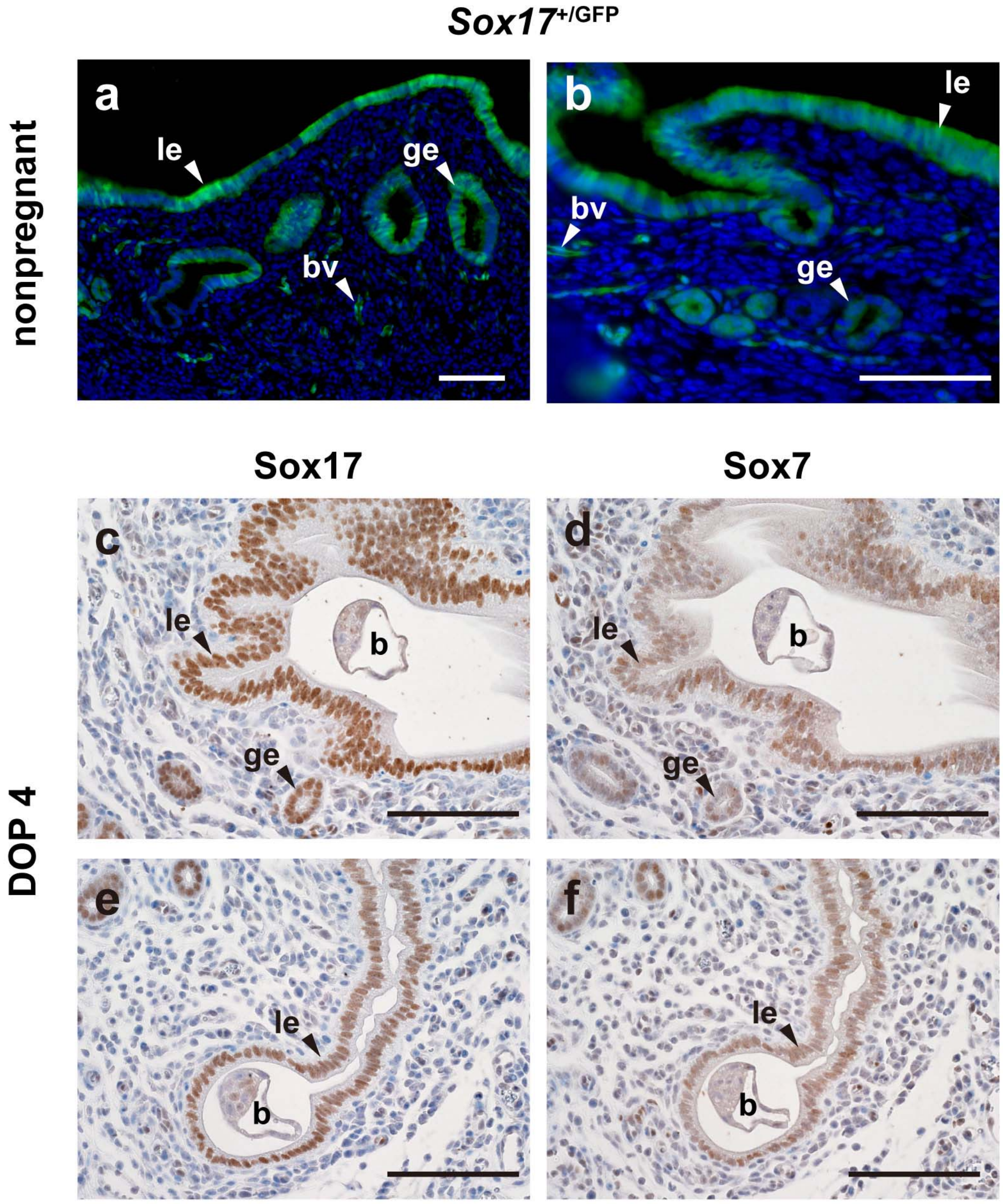

Supplementary Figure S1. Expression of Sox17 in the uterine endometrium.
(**a**, **b**) Fluorescence emitted from GFP is shown in green at lower magnification (**a**) and higher magnification (**b**). Nuclei are stained with Hoechst (blue). (**c–f**) Immunohistochemistry of Sox17 (**c**, **e**) and Sox7 (**d**, **f**) in the uterine endometrium at DOP 4. Crypt formation was more advanced in (**e**, **f**) than in (**c**, **d**). Abbreviations: le, luminal epithelium; ge, glandular epithelium; b, blastocyst; and bv, blood vessels. Scale bars, 100 μm.

a

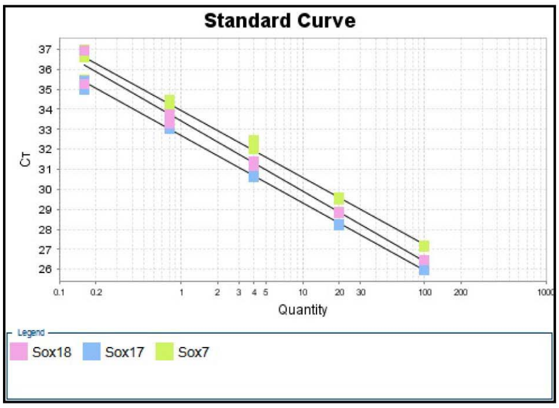

b

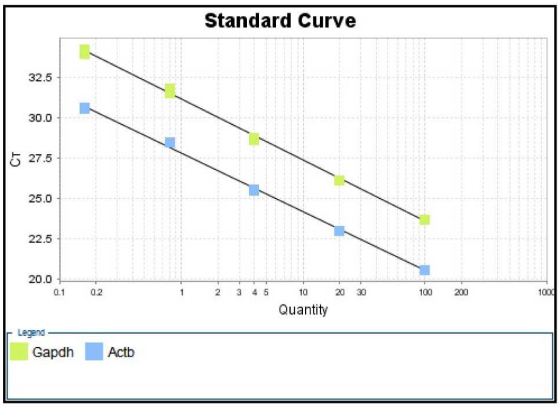

c Standard Curve

Target	Slope	Y-Inter	R^2	Eff%
Sox17	−3.366	32.671	0.998	98.205
Sox7	−3.33	33.932	0.988	99.673
Sox18	−3.512	33.41	0.988	92.633
Gapdh	−3.769	31.114	0.998	84.217
Actb	−3.64	27.809	0.998	88.239

d

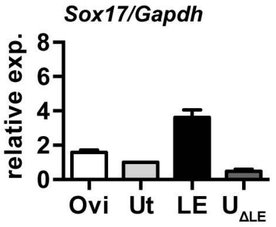

e

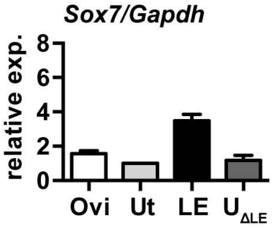

f

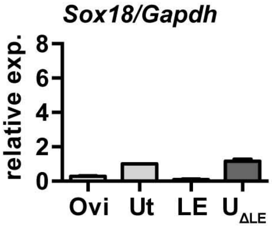

Supplementary Figure S2. Quantitative RT-PCR analysis of the expression of *Sox17*, *Sox7*, and *Sox18* mRNAs in the oviduct and uterine tissues.
(**a**–**c**) Standard curves of the genes examined by real-time PCR. Complementary DNA was reverse transcribed from total RNA isolated from the uterine tissue and used as a template. (**d**–**f**) Relative expression levels of *Sox17* (**d**), *Sox7* (**e**), and *Sox18* (**f**) transcripts in WT oviduct and uterine tissues at DOP 4, as determined by real-time RT-PCR. Relative expression levels ($2^{-\Delta\Delta CT}$) were analysed by the comparative C_T method (mean ± standard error of mean [SEM]). *Gapdh* was used as an internal control. The uterus was used as a calibrator.
